# Locus coeruleus integrity and left frontoparietal connectivity provide resilience against attentional decline in preclinical alzheimer’s disease

**DOI:** 10.1186/s13195-024-01485-w

**Published:** 2024-05-31

**Authors:** Jennifer Pahl, Prokopis C. Prokopiou, Elisenda Bueichekú, Aaron P. Schultz, Kathryn V. Papp, Michelle E. Farrell, Dorene M. Rentz, Reisa A. Sperling, Keith A. Johnson, Heidi I.L. Jacobs

**Affiliations:** 1 Athinoula A. Martinos Center for Biomedical Imaging, Department of Radiology, Massachusetts General Hospital, Harvard Medical School, Boston, MA USA; 2 Center for Alzheimer Research and Treatment, Department of Neurology, Brigham and Women’s Hospital, Harvard Medical School, Boston, MA USA; 3 Department of Neurology, Massachusetts General Hospital, Harvard Medical School, Boston, MA USA; 4 Department of Neurology, University Hospital RWTH Aachen, Aachen, Germany; 5 Gordon Center for Medical Imaging, Department of Radiology, Massachusetts General Hospital, Harvard Medical School, Boston, MA USA

**Keywords:** Alzheimer’s disease, Amyloid, Attention, Cognitive decline, Left frontoparietal network, Locus coeruleus, Resilience

## Abstract

**Background:**

Autopsy work reported that neuronal density in the locus coeruleus (LC) provides neural reserve against cognitive decline in dementia. Recent neuroimaging and pharmacological studies reported that left frontoparietal network functional connectivity (LFPN-FC) confers resilience against beta-amyloid (Aβ)-related cognitive decline in preclinical sporadic and autosomal dominant Alzheimer’s disease (AD), as well as against LC-related cognitive changes. Given that the LFPN and the LC play important roles in attention, and attention deficits have been observed early in the disease process, we examined whether LFPN-FC and LC structural health attenuate attentional decline in the context of AD pathology.

**Methods:**

142 participants from the Harvard Aging Brain Study who underwent resting-state functional MRI, LC structural imaging, PiB(Aβ)-PET, and up to 5 years of cognitive follow-ups were included (mean age = 74.5 ± 9.9 years, 89 women). Cross-sectional robust linear regression associated LC integrity (measured as the average of five continuous voxels with the highest intensities in the structural LC images) or LFPN-FC with Digit Symbol Substitution Test (DSST) performance at baseline. Longitudinal robust mixed effect analyses examined associations between DSST decline and (i) two-way interactions of baseline LC integrity (or LFPN-FC) and PiB or (ii) the three-way interaction of baseline LC integrity, LFPN-FC, and PiB. Baseline age, sex, and years of education were included as covariates.

**Results:**

At baseline, lower LFPN-FC, but not LC integrity, was related to worse DSST performance. Longitudinally, lower baseline LC integrity was associated with a faster DSST decline, especially at PiB > 10.38 CL. Lower baseline LFPN-FC was associated with a steeper decline on the DSST but independent of PiB. At elevated PiB levels (> 46 CL), higher baseline LFPN-FC was associated with an attenuated decline on the DSST, despite the presence of lower LC integrity.

**Conclusions:**

Our findings demonstrate that the LC can provide resilience against Aβ-related attention decline. However, when Aβ accumulates and the LC’s resources may be depleted, the functioning of cortical target regions of the LC, such as the LFPN-FC, can provide additional resilience to sustain attentional performance in preclinical AD. These results provide critical insights into the neural correlates contributing to individual variability at risk versus resilience against Aβ-related cognitive decline.

**Supplementary Information:**

The online version contains supplementary material available at 10.1186/s13195-024-01485-w.

## Background

Current pharmaceutical clinical trials in the Alzheimer’s disease (AD) field are targeting the earliest detectable pathologic markers with the goal of delaying and, if possible, preventing disease progression. AD neuropathologic change is characterized by an almost predictable topography of beta-amyloid (Aβ) and hyperphosphorylated tau (p-tau) accumulation [1, 2]. However, not all individuals with AD pathology demonstrate cognitive deficits or decline proportionally to their disease burden [3]. Identifying neural properties that provide this cognitive resilience in the face of pathology is critical for designing interventions that can effectively target and support brain mechanisms with the aim of delaying disease progression in early stages.

Several recent neuroimaging studies provided evidence that higher functional connectivity (FC) of the left frontoparietal network (LFPN) may provide reserve against cognitive decline in aging, as well as in sporadic and autosomal AD [4–7]. In individuals with elevated Aβ, the negative effect of elevated cerebrospinal fluid p-tau on cognitive decline was attenuated when LFPN-FC was higher [4]. While this was observed in individuals with evidence of AD neuropathologic change, the protective effect of LFPN-FC was also observed in earlier stages of cortical pathology. Higher LFPN-FC attenuated the negative effect of entorhinal tau on memory functioning, independent of Aβ-status [8]. The consistent involvement of the frontoparietal network in providing resilience is notable, as this network plays a key role in several cognitive functions, including attention, task shifting, and working memory [9, 10]. Greater distractibility has been associated with decreased activity and coherence in the frontoparietal network, which normally acts to reduce interference from distraction [11]. Attention is also one of the earliest affected cognitive domains in preclinical AD, and recent work suggested that processing speed or attention measures, not memory, can indicate the earliest Aβ-related cognitive changes [12, 13].

Connectivity within the frontoparietal network may also mitigate the adverse effect of tau aggregate accumulation in the locus coeruleus (LC), one of the earliest sites of tau deposition [1, 14–16]. Through its widespread noradrenergic (NA) projections, the LC can modulate multiple cognitive processes, including attention, memory, and cognitive control - functions that are also supported by frontal and parietal areas [9, 17–19]. Neuroimaging and pharmacological studies provided evidence that maintaining LC integrity is essential to preserve cognitive abilities and has been hypothesized to contribute to brain reserve capacity [20–22]. Recent work showed that FC between the LC and frontal regions declines with age and is associated with insufficient top-down attentional control [23]. Furthermore, Tomassini and colleagues (2022) demonstrated that lower LC structural integrity was associated with slower performance on a response inhibition task, and this relationship was mediated by greater prefrontal FC in older individuals [24]. While counterintuitive to a possible protective role of prefrontal FC, the variability in task performance and FC for the older group (65–88 years) was substantial, and it remains unknown whether these associations were modulated by covert AD pathology.

In this work, we aimed to examine whether LFPN-FC attenuates the impact of lower LC structural health on an attention-related measure that declines early in the course of AD. To this end, we investigated associations between baseline LC structural integrity, baseline LFPN-FC and attention functioning at baseline and longitudinally at varying levels of Aβ among well-characterized individuals from the Harvard Aging Brain Study (HABS) who were followed for up to 5 years.

## Methods

### Participants

A total of 142 participants from HABS who underwent LC imaging, resting state functional Magnetic Resonance Imaging (rs-fMRI), and 11 C-Pittsburgh Compound B (PiB)-Positron Emission Topography (PET), as well as annual cognitive assessments were included in the analysis. HABS is an ongoing observational study that aims to identify the earliest changes in molecular, functional, and structural imaging markers that signal the transition from normal to progressive cognitive decline along the trajectory of preclinical AD [25]. At study entry in HABS, participants had no history of medical or psychiatric disorders (a Geriatric Depression Scale (GDS) Score ≤ 10 [26]) and were cognitively normal as determined by a Clinical Dementia Rating Scale (CDR) Score = 0 [27], a Mini-Mental State Examination (MMSE) Score ≥ 26 [28], and normal performance within validated education-adjusted norms on the Logical Memory II delayed recall task [29]. Since LC imaging was added in HABS mid-study, the baseline for this study (*t* = 0) was defined as the time of each participant’s first LC imaging session. At the time of LC imaging, 13 participants had a CDR score > 0. In the present study, participants were included if rs-fMRI, PET, and cognitive data were available within one year of the LC imaging session. All imaging data included in this study constitute baseline (t = 0) measures. Cognitive data was collected from t = 0 (baseline) with follow-up of up to 5 years. The average difference between the MRI and PET imaging sessions was 0.18 (± 0.25) years.

### Imaging data

#### Structural MRI

All MRI data were collected at the Athinoula A. Martinos Center for Biomedical Imaging in Charlestown, MA on a 3T Siemens Tim-Trio scanner with a 12-channel phased-array head coil. Head motion was minimized with foamed padding placed around the head. Structural T1-weighted images were acquired as magnetization-prepared rapid acquisition gradient echo (MPRAGE). The following acquisition parameters were used: repetition time (TR) = 2300 ms; echo time (TE) = 2.95 ms; inversion time (TI) = 900 ms; flip angle = 9°; resolution = 1.05 ✕ 1.05 ✕ 1.20 mm. A 2D T1-weighted turbo-spin-echo (TSE) sequence with additional magnetization transfer contrast was used to visualize the LC (TR = 743 ms; TE = 16 ms; flip angle = 180°; six slices; four online averages; 0.4 ✕ 0.4 ✕ 3.0 mm resolution). The short acquisition time minimized motion artifacts, which is critical given the LC’s size and close proximity to the fourth ventricle. Data were processed using FreeSurfer (FS) 6 (http://surfer.nmr.mgh.harvard.edu) using the software package’s default, automated reconstruction protocol as described in detail elsewhere [30]. FS-automated segmentation results were manually inspected using its visualization tool Freeview and, if necessary, edited.

#### Resting-state fMRI

Whole-brain rs-fMRI data were acquired using a gradient-echo planar imaging (EPI) sequence sensitive to blood-oxygenation level-dependent (BOLD) contrast, aligned parallel to the anterior/posterior commissure. The following parameters were used: TR = 3000 ms; TE = 30 ms; flip angle = 85°; field of view = 216 ✕ 216 mm; matrix = 72 ✕ 72; and 3 ✕ 3 ✕ 3 mm voxels. In total, 124 volumes were acquired in each of two 6:12-minute runs for a total of 12.24 min. Participants were instructed to lie still, remain awake and keep their eyes open.

All data were processed using the Oxford Center for Functional Magnetic Resonance Imaging of the Brain Software Library (FSL; Version 5.0.7) [31]. To allow for T1 equilibration, the first five dummy volumes of each run were excluded. Data preprocessing steps included: brain extraction, slice-time correction, motion realignment and normalization to the 2 mm^2^ Montreal Neurological Institute (MNI) – 152 standard template using FSL’s non-linear registration tool (FNIRT) [32]. We applied spatial smoothing at a 5 mm FWHM Gaussian kernel. Subsequently, further denoising was performed by regressing out the realignment parameters (plus first derivatives and their squares) and by applying high pass filtering at 0.005 Hz.

Rs-FC values were calculated in two steps. First, the BOLD-fMRI data were concatenated in time across all participants and decomposed into statistically independent spatial components of underlying brain activity (group-level functional networks) with probabilistic independent component analysis (pICA) using FSL’s Multivariate Exploratory Linear Decomposition into Independent Component (MELODIC) tool [33]. We identified group-level spatial maps for the left and right frontoparietal networks (LFPN and RFPN, respectively). Second, subject-specific contributions to each of these group-level spatial maps were calculated using dual regression [34]. This involved using the group maps in a linear model fit, yielding matrices describing subject-specific temporal components that were subsequently regressed against the individual BOLD-fMRI data to obtain voxel-wise regression z-score maps describing the functional networks within each participant [35]. Lastly, subject-specific network FC values were obtained for the RFPN and LFPN as the average dual-regression z-score value across all voxels exhibiting a z > 4.5. For the purpose of this work, LFPN-FC was used as a main predictor, while RFPN-FC was used as a control network to determine the specificity of our results.

#### Identification and quantification of LC integrity

LC signal intensity was calculated from the 2D T1-TSE images as previously described [15]. In brief, four equidistant boxes were initially defined on the 0.5 mm MNI template covering the LC region and the rostral pontine tegmentum (reference region) bilaterally. These boxes were used as boundary regions to guide the search for intensities related to the structure of interest and to remove any possible experimenter bias in identifying the LC. Subsequently, these boundary regions were warped to each individual LC scan in a two-step procedure. First, the MNI template was registered to the individual T1w structural image using non-linear diffeomorphic registration and then to the individual LC scan space using linear, rigid-body registration. To ensure that the LC intensity values can be compared across participants, each slice in the LC scan was normalized with respect to the reference region. Finally, LC intensity was determined as the maximum (across 30 iterations) of the average between all voxels within a cluster of connected voxels exhibiting the highest intensity values. Consistent with existing literature, LC intensity was measured using 5 continuous voxels with the highest intensities and will be referred to as LC integrity, potentially reflecting neuronal density of the LC and correlating strongly with tau accumulation [15].

#### Positron emission tomography

PiB-PET data were collected at Massachusetts General Hospital on a Siemens/ CTI ECAT HR + scanner as previously reported [36]. PiB-PET images were acquired with an 8.5–15 mCl bolus injection with a 1-hour dynamic acquisition over 69 volumes (12 ✕ 15 s, 57 ✕ 60 s). PET images were reconstructed using standard correction procedures [37]. Each frame was evaluated to verify adequate count statistics, and an automated frame-to-frame realignment algorithm was applied and visually checked to correct for motion artifacts.

Individual PiB-PET data was expressed as the distribution volume ratio (DVR) using the Logan graphical method and cerebellar grey as the reference region, applied over 40- to 60-minute post-injection integration intervals. Partial volume correction (PVC) was performed using a geometrical transfer matrix (GTM) method, which assumed an isotropic 6 mm point spread function. Neocortical PiB retention was evaluated as the average uptake in a large aggregate region, consisting of areas within the frontal, lateral and retrosplenial (FLR) cortices. Classification into elevated (PiB+) versus low Aβ (PiB-) groups (DVR-PVC) was ascertained based on a Gaussian mixture modeling approach identifying a PiB cutoff value of 1.324 (equal to Centiloid (CL): 18.49) [38].

### Cognitive measures

To evaluate attentional performance at baseline and longitudinally, we used the Wechsler Adult Intelligence Scale-Revised Digit Symbol Substitution Test (DSST) [13, 29]. To determine the specificity of the results, we also included tests of episodic memory functioning sensitive to detect preclinical AD, the Free and Cued Selective Reminding Test total (FCSRT, delayed total, free and cued scores) measured during the same visits [39, 40]. In total, DSST and FCSRT scores were available for all 142 participants, and longitudinal data consisted of 457 observations (observations per year: 1st = 146, 2nd = 138, 3rd = 100, 4th = 60, 5th = 13).

### Statistical analysis

Statistical analyses were performed using the statistical software R (version 4.1.2). The statistical significance threshold was set at *p* < 0.05. All data were inspected for violation of normality and influential cases. Due to the presence of potential outliers, all analyses were performed using robust linear regression using the Huber M-estimator. First, we related baseline LC integrity or LFPN-FC independently with cross-sectional DSST scores, including age, sex, and years of education as covariates. Then, we examined the effect modification of PiB (DVR-PVC) on cross-sectional DSST scores by interacting PiB with either LC integrity or LFPN-FC at baseline.

Next, we examined longitudinal associations by relating baseline LC integrity or LFPN-FC to longitudinal DSST scores using robust linear mixed effects (RLME) models. In these models, DSST was the time-varying outcome measure, LC integrity or LFPN-FC were included independently as fixed effects along with age, sex, and years of education, and interacted with time. We further included a random intercept for participants. Random slope models (time) were considered but did not converge or demonstrated an inferior fit based on the AIC and BIC model fit indices. Similar to the aforementioned cross-sectional models, we also examined the effect modification by PiB (DVR-PVC) on the relationship between longitudinal DSST change and baseline LC integrity or LFPN-FC and, if applicable, performed post-hoc floodlight analyses to identify the range of PiB values at which the relationship between LC integrity or LFPN-FC with DSST change became significant. For all RLME models, parameter estimation was performed using maximum likelihood estimation.

We then aimed to examine whether LC integrity and LFPN-FC at baseline act synergistically on DSST decline in the setting of Aβ pathology (i.e., PiB load). Given the complexity of four-way interaction terms, we extracted the individual DSST slopes from the LME model and examined the effect of the three-way interaction between LC integrity, LFPN-FC and PiB (DVR-PVC) at baseline on DSST slopes using robust linear regression. Age, sex, and years of education were included as covariates. These analyses were also followed up with floodlight analyses. The individual DSST slopes were extracted from a longitudinal robust linear mixed effects model, including the participants’ longitudinal (time-varying) DSST scores as the outcome variable, time as fixed effect, random slopes for time, and random intercepts for participants.

As part of our control analyses, we repeated all abovementioned analyses using the FCSRT total recall score as a control cognitive test (instead of the DSST) and using the RFPN as a control network (i.e. RFPN-FC) instead of the LFPN. In addition, we performed sensitivity analyses to ensure that the 13 individuals with a CDR > 0 at t = 0 were not driving our results. We thus repeated all analyses including baseline CDR as an additional covariate in our models. The results of our control and sensitivity analyses will be described in the main text, while all related figures and tables can be found in the supplementary material. No multiple comparison correction was applied since this was a hypothesis-driven research including control analyses. For clarification, please find the general models of the performed analyses below:

Baseline analyses (robust linear regression models):

1. *Baseline cognition ~ LC integrity (or FC) + covariates*.
2. *Baseline cognition ~ LC integrity (or FC)× PiB + covariates*.

Longitudinal analyses (robust mixed effect models):

3. *Longitudinal cognition ~ LC integrity (or FC) × time + covariates × time*.
4. *Longitudinal cognition ~ LC integrity (or FC) × PiB × time + covariates × time*.

*Random effects: random intercepts = participants, no random slopes.*

Synergistic effects models (robust linear regression analyses using cognitive slopes):

5. *Cognitive slopes ~ LC integrity × FC × PiB + covariates*.

#### Covariates

age, sex, and years of education (and baseline CDR as sensitivity analysis).

#### Cognition

DSST scores (or FSCRT scores in the control analyses).

#### FC

functional connectivity of the LFPN (or RFPN in the control analyses).

## Results

### Participants

The demographics of the 142 participants are provided in Table 1. The number of available annual cognitive assessments ranged from 1 to 5 years. At the time of the LC imaging session (baseline; t = 0), 12 individuals had progressed to a CDR of 0.5 (8.5%) and one individual to a CDR of 1 (0.7%). Based on the previously defined PiB cutoff value of 1.324 (DVR-PVC), 45 (31.7%) participants were classified as having elevated aβ (PiB + status) at baseline. Based on age and Aβ levels, we can deduct from the Braak staging framework that the majority of our individuals will have at least Braak stage II pathology [1].

**Table 1:** Characteristics of participants at baseline neuropsychological evaluation

*n* (total)	142
Age (years)	74.5 [69.88, 82.12]
Sex, No. (%) = F	89 (62.7)
Education (years)	16 [14, 18]
MMSE (score)	29 [28, 30]
CDR, No. (%) = 0, 0.5, 1	129 (90.8), 12 (8.5), 1 (0.7)
DSST (score)	47 [37, 57.75]
NP follow-up (years)	1.47 [0, 2.26], max = 4.96
PiB status, No. (%) = PiB +	45 (31.7)
PiB, DVR FLR (PVC)	1.236 [1.167, 1.546]
LC integrity (a.u.)	1.318 [1.293, 1.346]
LFPN-FC (a.u.)	0.056 [0.038, 0.084]

Note. Data are presented as medians and [interquartile ranges (IQRs)] for continuous variables and proportions. Abbreviations: a.u. = Arbitrary units, CDR = Clinical Dementia Rating, DSST = Digit Symbol Substitution Test, DVR = distribution volume ratio, FC = functional connectivity, FLR = frontal, laterotemporal and retrosplenial cortices, F = female, LC = locus coeruleus, LFPN = left frontoparietal network, MMSE = Mini-Mental Status Examination, NP = neuropsychological evaluation, PVC = partial volume corrected, PiB = Pittsburgh Compound-B.

### Lower LFPN-FC is related to worse attentional performance at baseline

**Fig. 1:**
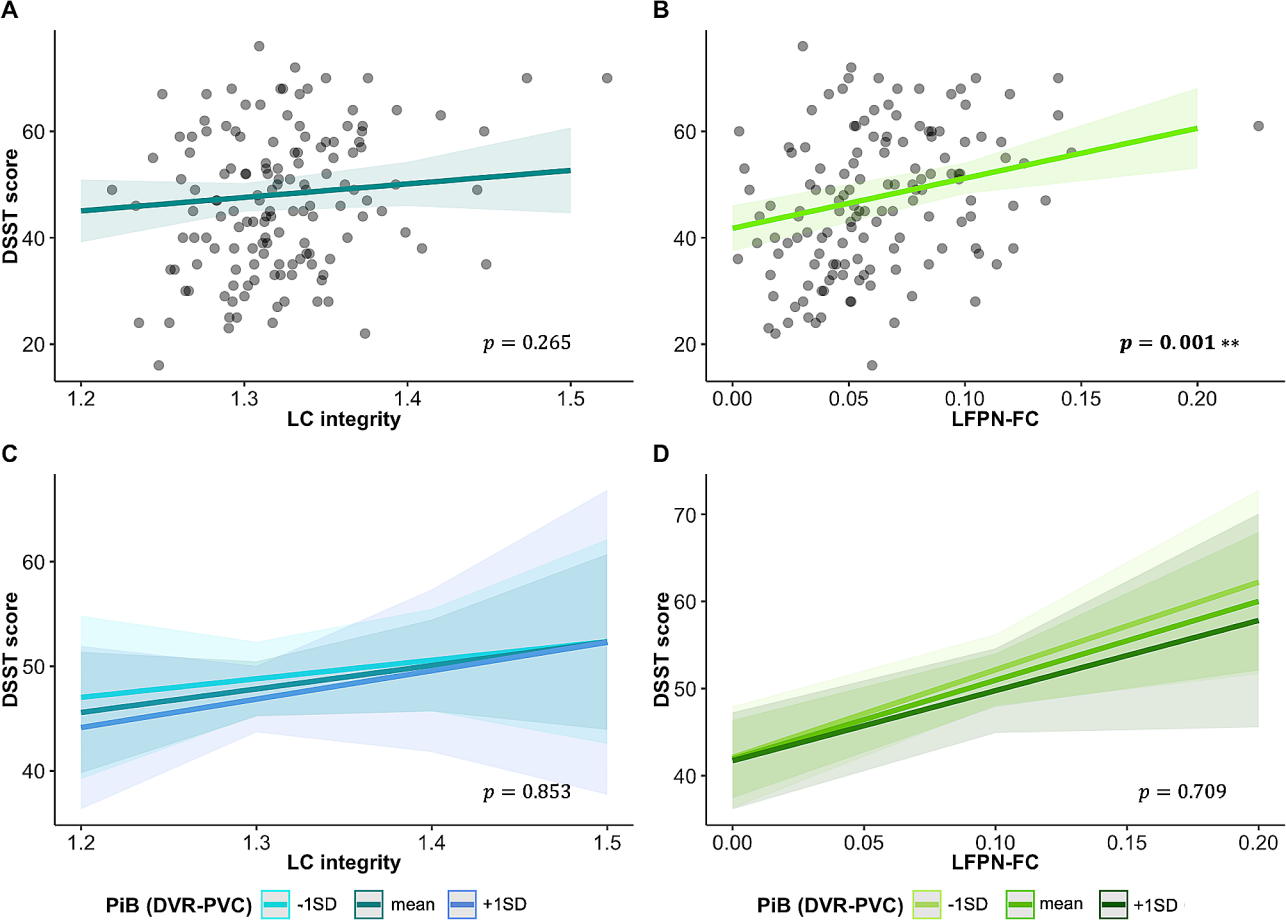
Effect of LC integrity and LFPN-FC on DSST scores at baseline *Note*. Visualization of the association between baseline LC integrity (in blue) and baseline DSST scores (**A**) at different PiB levels (**C**); and of the association between baseline LFPN-FC and baseline DSST scores (**B**) at different PiB levels (**D**) in green. The estimated marginal means of the interaction terms were plotted at the mean and ± 1 SD for PiB load, but analyses were performed continuously. Shaded regions represent the 95% confidence interval. The units for LC integrity and LFPN-FC are arbitrary. *Abbreviations*: DSST = Digit Symbol Substitution Test, DVR = distribution volume ratio, FC = functional connectivity, LC = locus coeruleus, LFPN = left frontoparietal network, PVC = partial volume corrected, PiB = Pittsburgh Compound-B, SD = standard deviation

There was no significant association between DSST performance and LC integrity at baseline (B = 25.23, t_137_ = 1.12, *p* = 0.265, 95% confidence interval (CI) [-19.38, 69.84]; Fig. 1A), also not when interacting LC integrity with PiB (B = 7.47, t_135_ = 0.10, *p* = 0.853, 95%CI [-147.39, 162.33]; Fig. 1C). Lower LFPN-FC was associated with worse performance on the DSST at baseline (B = 94.06, t_137_ = 3.39, *p* = 0.001, 95%CI [39.21, 148.91]; Fig. 1B), but this was not modified by PiB (B= -22.31, t_135_=-0.37, *p* = 0.709, 95%CI [-140.38, 95.77]; Fig. 1D).

We then repeated the analyses using baseline FCSRT total recall scores as part of our control analyses. At baseline, better performance on the FCSRT was associated with greater LC integrity (B = 35.01, t_137_ = 2.37, *p* = 0.019, 95%CI [5.80, 64.22]; S1A), particularly at elevated PiB levels (B = 81.59, t_135_ = 2.31, *p* = 0.022, 95%CI [11.80, 151.38]; Figure S1C). No significant association between performance on the FCSRT and LFPN-FC was observed at baseline (B= -14.32, t_137_= -0.72, *p* = 0.473, 95%CI [-53.68, 25.03]; Figure S1B), nor when LFPN-FC was interacted with PiB (B= -9.38, t_135_= -0.21, *p* = 0.835, 95%CI [-98.32, 79.55]; Figure S1D). Further, also as part of our control analyses, we repeated these models using RFPN-FC as a control network (instead of LFPN-FC) and found no association between RFPN-FC and DSST performance at baseline (B= -16.90, t_137_= -0.74, *p* = 0.460, 95%CI [-62.02, 28.21]), and no interaction between RFPN-FC and PiB (B= -24.63, t_135_= -0.43, *p* = 0.67, 95%CI [-138.66, 89.40]; Figure S2A). As part of the sensitivity analyses, the abovementioned analyses were repeated including baseline CDR as an additional covariate into the models. The results of baseline LC integrity and LFPN-FC, as well as their interaction with PiB, on cross-sectional DSST scores remained unchanged (see Table S1).

### Lower baseline LC integrity and LFPN-FC are related to a steeper decline in attention

**Fig. 2:**
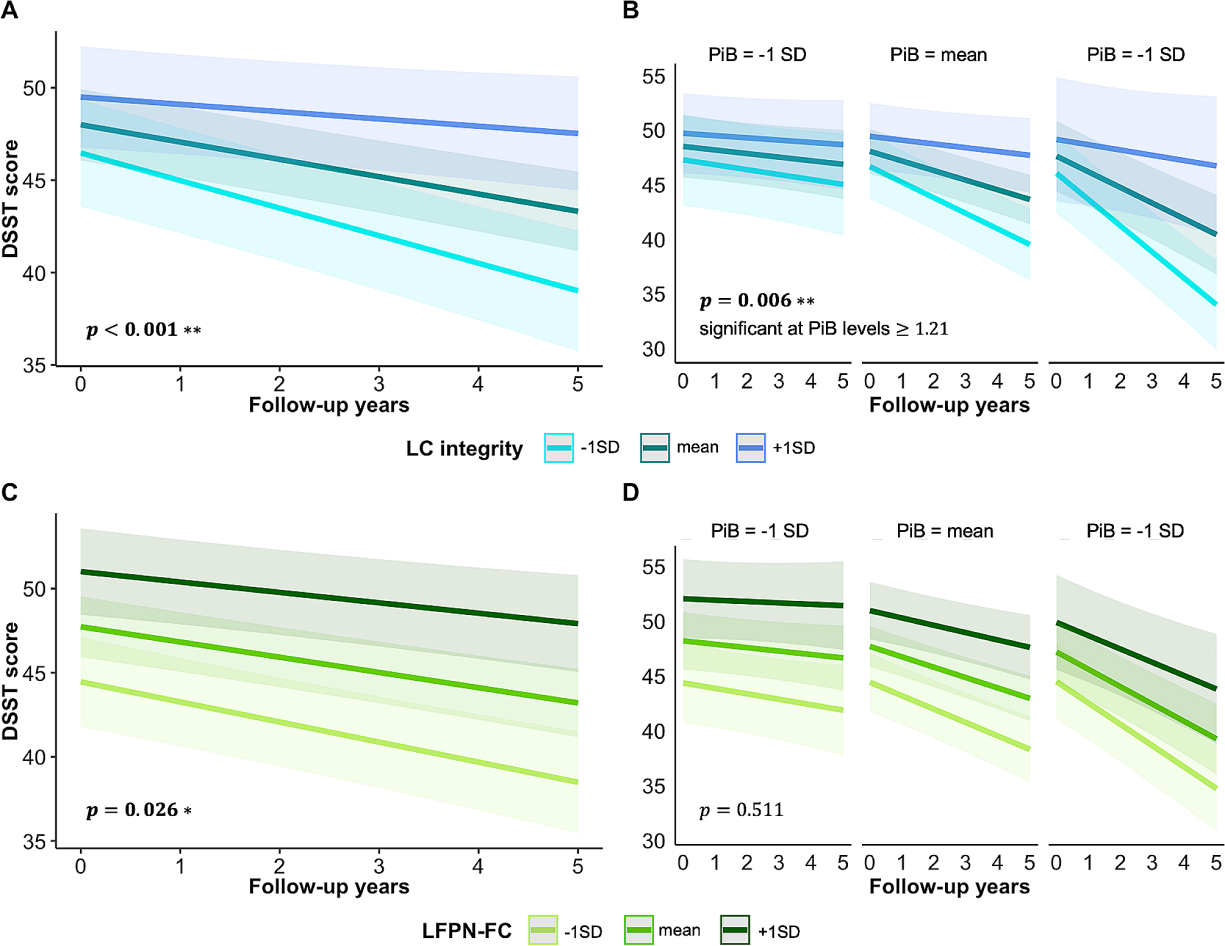
Effect of baseline LC integrity and LFPN-FC on longitudinal DSST scores *Note*. Visualization of the association between baseline LC integrity (in blue; **A**) or LFPN-FC (in green; **C**) on DSST decline over time, plotted at different levels of PiB burden (**B** and **D**). The estimated marginal means of the interaction terms were plotted at the mean and ± 1 SD for PiB load, but analyses were performed continuously. Shaded regions represent the 95% confidence interval. The units for LC integrity and LFPN-FC are arbitrary. *Abbreviations*: DSST = Digit Symbol Substitution Test, DVR = distribution volume ratio, FC = functional connectivity, LC = locus coeruleus, LFPN = left frontoparietal network, PVC = partial volume corrected, PiB = Pittsburgh Compound-B, SD = standard deviation

We observed that lower baseline LC integrity was related to a steeper decline on the DSST over time (B = 11.55, t_299_ = 3.52, *p* < 0.001, 95%CI [5.12, 17.98]; Fig. 2A), particularly in individuals with elevated PiB levels (B = 19.76, t_295_ = 2.73, *p* = 0.006, 95%CI [5.57, 33.95]; see Fig. 2B). Floodlight analyses revealed that the association between LC integrity and DSST decline becomes significant at PiB levels above or equal to 1.21 DVR-PVC (10.38 CL), which is below the HABS PiB positivity cut-off of 1.324. Furthermore, our data demonstrate that at higher values of baseline LC integrity, DSST decline is attenuated, even in the face of elevated Aβ (Fig. 2B). Similarly, greater baseline LFPN-FC was associated with increasing DSST scores over time (B = 9.11, t_299_ = 2.23, *p* = 0.026, 95%CI [1.10, 17.13]; Fig. 2C), but was not modulated by PiB (B = 5.84, t_295_ = 0.66, *p* = 0.511, 95%CI [-11.57, 23.25]; see Fig. 2D).

We then repeated the analyses using the FCSRT total recall score as the control cognitive outcome measure and the RFPN as a control network, separately. Baseline LC integrity was not related to decline over time on the FCSRT (B= -1.85, t_299_= -0.55, *p* = 0.582, 95%CI [-8.45, 4.74]; Figure S3A), nor when LC integrity was interacted with PiB (B = 11.83, t_295_ = 1.55, *p* = 0.122, 95%CI [-3.18, 26.83]; Figure S3B). Similarly, baseline LFPN-FC was not associated with a decline on the FCSRT (B = 5.32, t_299_ = 1.26, *p* = 0.208, 95%CI [-2.97, 13.62]; See Figure S3C), also not when LFPN-FC was interacted with PiB (B = 12.77, t_295_ = 1.36, *p* = 0.175, 95%CI [-5.70, 31.25], see Figure S3D). Furthermore, no significant associations between DSST decline and baseline RFPN-FC (control network) were observed (B = 0.46, t_299_=-0.145, *p* = 0.885, CI [-5.72, 6.63]), nor when RFPN-FC was interacted with PiB (B= -5.38, t_295_= -0.673, *p* = 0.501, 95%CI [-21.06, 10.29]; Figure S2B). When controlling for baseline CDR, the effects of baseline LC integrity and LFPN-FC, as well as their interaction with PiB, on DSST decline over time were similar (see Table S1).

### Greater baseline LFPN-FC counteracts lower LC integrity against attentional decline over time in individuals with greater Aβ cortical deposition

**Fig. 3:**
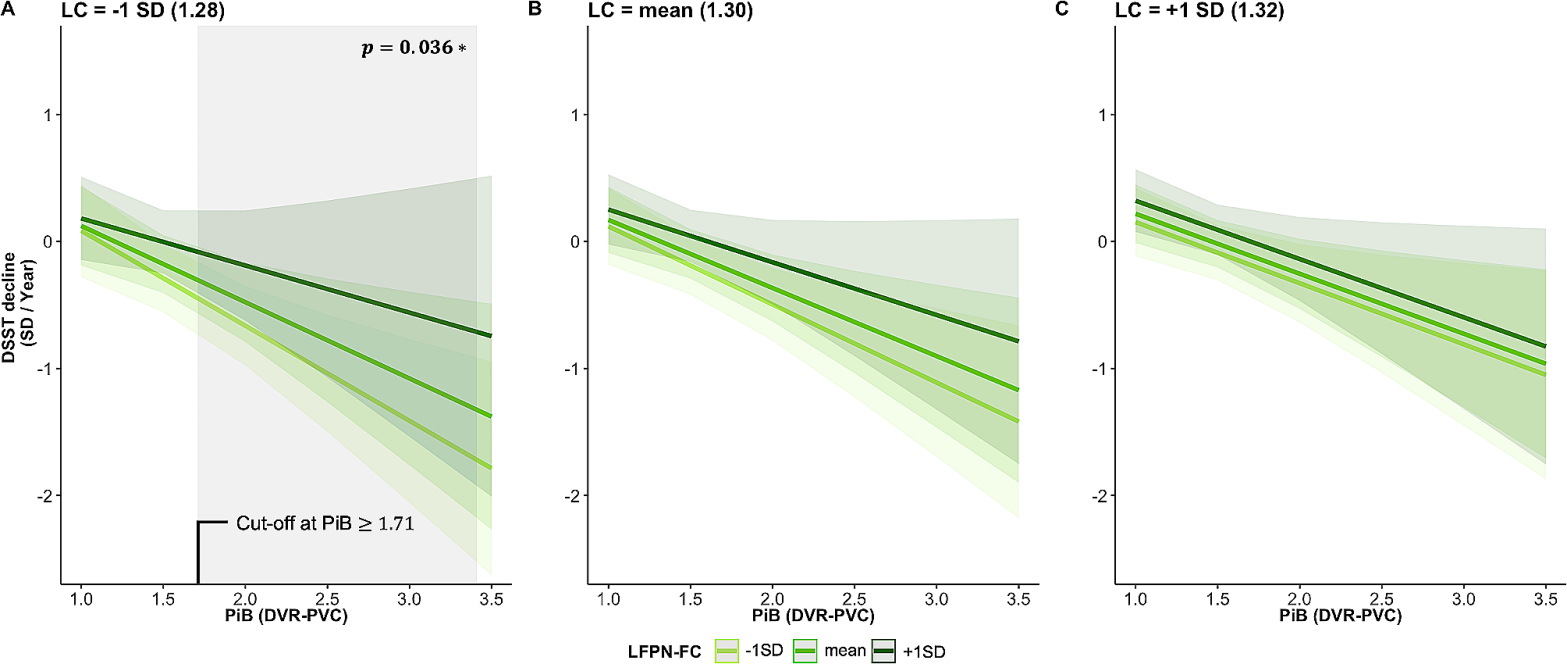
Synergistic effect of baseline LC integrity, LFPN-FC and PiB deposition on DSST decline over time *Note*. Visualization of the synergistic association between LC integrity, LFPN-FC and PiB load at baseline on DSST decline over time, plotted at different levels of LC integrity and LFPN-FC. The estimated marginal means of the interaction terms were plotted at the mean and ± 1 SD for LC and LFPN-FC, but analyses were performed continuously. Shaded regions in green represent the 95% confidence interval. Shaded in grey is the area for which the interaction effect is significant (Johnson-Neyman interval). The units for LC integrity and LFPN-FC are arbitrary. *Abbreviations*: DSST = Digit Symbol Substitution Test, DVR = distribution volume ratio, FC = functional connectivity, LC = locus coeruleus, LFPN = left frontoparietal network, PVC = partial volume corrected, PiB = Pittsburgh Compound-B, SD = standard deviation

Overall, our findings suggest that both, greater LC integrity and LFPN-FC at baseline, have a protective effect on DSST decline, also in the context of elevated PiB levels. Subsequently, we investigated whether LC integrity and LFPN-FC at baseline can act synergistically in attenuating DSST decline at higher PiB values. Lower baseline LC integrity and lower LFPN-FC are associated with faster PiB-related DSST decline, but higher levels of LFPN-FC attenuate the negative effect of lower LC integrity on DSST decline, even at higher levels of PiB deposition (B= -222.56, t_131_= -2.12, *p* = 0.036, 95%CI [-430.52, -14.61]; Fig. 3). Floodlight analyses revealed that this association emerges at high PiB values, equal or higher than 1.71 DVR-PVC (46 CL). Control analyses using the FCSRT showed no significant interaction between LC integrity, LFPN-FC and PiB deposition at baseline on FCSRT scores over time (B= -37.33, t_131_=, *p* = 0.486, 95%CI [-142.91, 68.25]; Figure S4A). Similarly, no interaction effect was observed when replacing LFPN-FC by RFPN-FC as a control network (B= -51.54, t_131_=, *p* = 0.628, 95%CI [-261.36, 158.28], Figure S4B). Our sensitivity analyses, including baseline CDR as covariate, showed similar associations between LC integrity, LFPN-FC and PiB load at baseline on DSST decline (see Table S1).

## Discussion

Understanding the neural correlates contributing to resilience against cognitive decline in the face of AD pathology is critical to the current goal of developing preventive interventions in our field. A substantial body of evidence demonstrated that higher LFPN-FC can counteract the negative downstream effects of cortical AD pathology on cognition in preclinical and prodromal stages of AD. Here, guided by previous studies correlating structural LC integrity with cognitive reserve in older individuals, we examined the contribution of the LC – one of the first regions affected by tau pathology in AD – to cognitive resilience in preclinical AD. Consistent with the existing literature, we observed that lower baseline LFPN-FC was associated with a decline in attention, independent of cortical Aβ deposition. Lower baseline LC integrity was associated with attentional decline, particularly when Aβ is elevated. Importantly, our study extended these findings by showing that the negative effect of lower LC integrity on Aβ-related attention decline can be mitigated by higher LFPN-FC. Crucially, accounting for participants’ baseline clinical dementia rating score did not change the nature of our results, further emphasizing the relevance of our findings for preclinical disease stages. Our findings highlight the important role of the LC in regulating attention and its critical interactions with network dynamics to modulate cognitive performance. It further shows that when LC structural health is impacted, the capacity of the target cortical networks to maintain communication can attenuate the cognitive sequela even under elevated AD pathology.

Previous studies examining the neural correlates of resilience in AD uncovered the importance of the LFPN, specifically the left frontal cortex hub [5, 7]. While these studies reported that higher LFPN-FC was associated with attenuated cognitive decline in attention and executive functioning in preclinical and prodromal AD, they also – consistent with our findings – indicated that LFPN-FC curbed cognitive decline in the setting of early tau pathology, independent of Aβ [8]. While we did not have tau PET data available on all these individuals, based on age and Aβ levels, we can deduce from the Braak staging framework that the majority of our individuals will have at least Braak stage II pathology [1].

Understanding the role of the LC in cognition and AD is an emerging topic of interest, and the LC has been described as a biomarker of cognitive reserve as well as of early AD risk [15, 21, 22]. Recent work demonstrated that LC structural integrity correlated with key indicators of cognitive reserve in older individuals and together contributed to attentional functioning [21].The role of the LC in cognitive reserve and resilience stems from decades of evidence on how the LC orchestrates flexibility and precision across the entire brain due to its widespread projections [17–19, 41]. These widespread innervations allow it to release NE, act upon adrenoreceptors and modulate neuronal firing and network connectivity. Furthermore, the LC modulates many cognitive processes, including novelty detection, learning, and attention [20], plays an essential role in neuroprotection and neuroinflammation [42], and is resilient to neuronal death despite its early involvement in AD pathology [1, 14].

Similarly, we recently demonstrated that while lower LC structural integrity and novelty-related FC were associated with faster Aβ-related decline, there were also several participants who did not exhibit cognitive decline under higher Aβ levels if they also exhibited higher FC levels [15, 43, 44]. This suggests that even though the LC is affected by tau pathology, other compensatory mechanisms arise that can upregulate cortical dynamics, potentially including increased firing rates, lower NE uptake, higher adrenoreceptor expression or sprouting [45, 46]. Consistent with these hypotheses, we found that lower LC structural integrity was associated with steeper Aβ-related attentional decline emerging from 10.38 CL, but that attentional decline was attenuated under higher LC integrity levels, despite the presence of elevated Aβ. Importantly, these associations were specific to attention, as lower LC integrity was not associated with memory decline, also not under elevated Aβ. These observations substantiate earlier findings in our group that attentional processes are amongst the earliest cognitive domains affected in preclinical AD [13]. This is important since attention is crucial to facilitate multiple cognitive processes, including learning and episodic memory [9, 47].

Notably, our findings extend these observations by showing that when Aβ accumulates (starting from 44CL) and LC integrity is low, higher LFPN-FC can still mitigate Aβ-related attentional decline. This is consistent with the protective role of LFPN-FC in aging and dementia [4–7]. Previous human [48] and animal work [49] demonstrated that as LC pathology progresses, the LC undergoes a series of significant morphological alterations, including dendritic atrophy and axonal shrinkage [50], reducing the LC’s capacity for optimal NA neurotransmission and cognitive function [51–53]. Recent animal work in rats exhibiting developing endogenous LC tau (TgF344-AD) displayed loss of LC axons. But these morphological changes coincided with compensatory mechanisms in its target regions, such as increased β-adrenergic receptor signaling and preserved learning [54, 55]. In fact, postmortem work reported preserved to heightened expression of postsynaptic α-adrenergic receptors in the prefrontal cortex of patients with AD [56]. We speculate that similar compensatory mechanisms at the target regions of the LC, even when LC integrity is declining and Aβ is rising, could enhance LFPN network efficiency [6], and support the maintenance of cognitive flexibility in the preclinical and prodromal phases of AD. Future studies that are focused on investigating later stages of the disease are needed to determine when LFPN-FC is no longer able to compensate for cumulating pathologic changes. Thus, while LC structural integrity proves pivotal as a prognostic measure evaluating the risk and progression of cognitive decline, targeting LFPN-FC emerges as another potential intervention strategy, particularly when LC integrity is compromised and Aβ levels are rising.

### Limitations

Our study has several limitations. First, generalizability of our findings may be limited as our HABS sample is highly educated, has an above-average IQ and is predominantly female. Furthermore, the DSST does not only capture attention, but also depends on the individual’s working memory capacity and processing speed. Therefore, future studies should complement and extend these findings by using different measures that can capture the broad realm of attentional functioning. Further, due to the recent introduction of LC imaging in HABS, we were not yet able to relate longitudinal changes in LC integrity, and changes in network connectivity, to AD-related cognitive decline. Therefore, our cross-sectional imaging data cannot provide any inferences on the temporal ordering or directionality of the investigated processes. Lastly, this work used a threshold of *p* < 0.05 for statistical significance and our findings should thus be interpreted with caution.

## Conclusion

In conclusion, we show that lower LC structural health contributes to AD-related attentional decline, while lower LFPN-FC contributes to attentional functioning in healthy and pathological aging. While preserving LC integrity may be specifically crucial during the at-risk and preclinical stages, LFPN-FC can confer resilience against attentional decline in the face of elevated Aβ and poor LC integrity. Our work highlights that variation in the interaction between the LC and cortical networks may explain individual differences in risk of or resilience against cognitive decline in AD.

### Electronic supplementary material

Below is the link to the electronic supplementary material.

Supplementary Material 1

Supplementary Material 2

Supplementary Material 3

Supplementary Material 4

Supplementary Material 5

## Data Availability

The Harvard Aging Brain Study project is committed to publicly releasing its data. Baseline data is already available online at http://nmr.mgh.harvard.edu/lab/harvardagingbrain/data. Follow-up data of the Harvard Aging Brain Study data, including the data used in this manuscript, will be made public to the research community, and data until year 5 is currently available by request, pending approval of a data request and agreement to abide by the Harvard Aging Brain Study online data use agreement.

## Abbreviations

Aβ: Beta-amyloid
AD: Alzheimer’s disease
BOLD: Blood-oxygen level-dependent
CDR: Clinical Dementia Rating
DSST: Digit Symbol Substitution Test
DVR: Distribution volume ratio
FC: Functional connectivity
FCSRT: Free and Cued Selective Reminding Test
FLR: Frontal, lateral and retrosplenial cortices
FPN: Frontoparietal network
FS: FreeSurfer
FSL: Functional Magnetic Resonance Imaging of the Brain Software Library
fMRI: Functional magnetic resonance imaging
FWHM: Full width half maximum
GDS: Geriatric Depression Scale
GTM: Geometrical transfer matrix
HABS: Harvard Aging Brain Study
IQ: Intelligence quotient
LC: Locus coeruleus
LFPN: Left frontoparietal network
MELODIC: Multivariate Exploratory Linear Decomposition into Independent Component
MMSE: Mini-Mental State Examination
MNI: Montreal Neurological Institute
MRI: Magnetic Resonance Imaging
NA: Noradrenaline, noradrenergic
NP: Neuropsychological assessment
PET: Positron emission topography
PiB: Pittsburgh Compound-B
pICA: Probabilistic independent component analysis
p-tau: Hyperphosphorylated tau
PVC: Partial volume correction
RFPN: Right frontoparietal network
rs-FC: Resting state functional connectivity
rs-fMRI: Resting-state functional magnetic resonance imaging
SUVR: Standardized uptake value ratio
TE: Echo time
TI: Inversion time
TR: Repetition time
TSE: Turbo-spin-echo
