# Supplementary figures and images for: Locus coeruleus integrity and left frontoparietal connectivity provide resilience against attentional decline in preclinical alzheimer’s disease
Source: Alzheimers Res Ther. 2024 May 31;16:119. doi: 10.1186/s13195-024-01485-w (PMC11140954; doi:10.1186/s13195-024-01485-w)

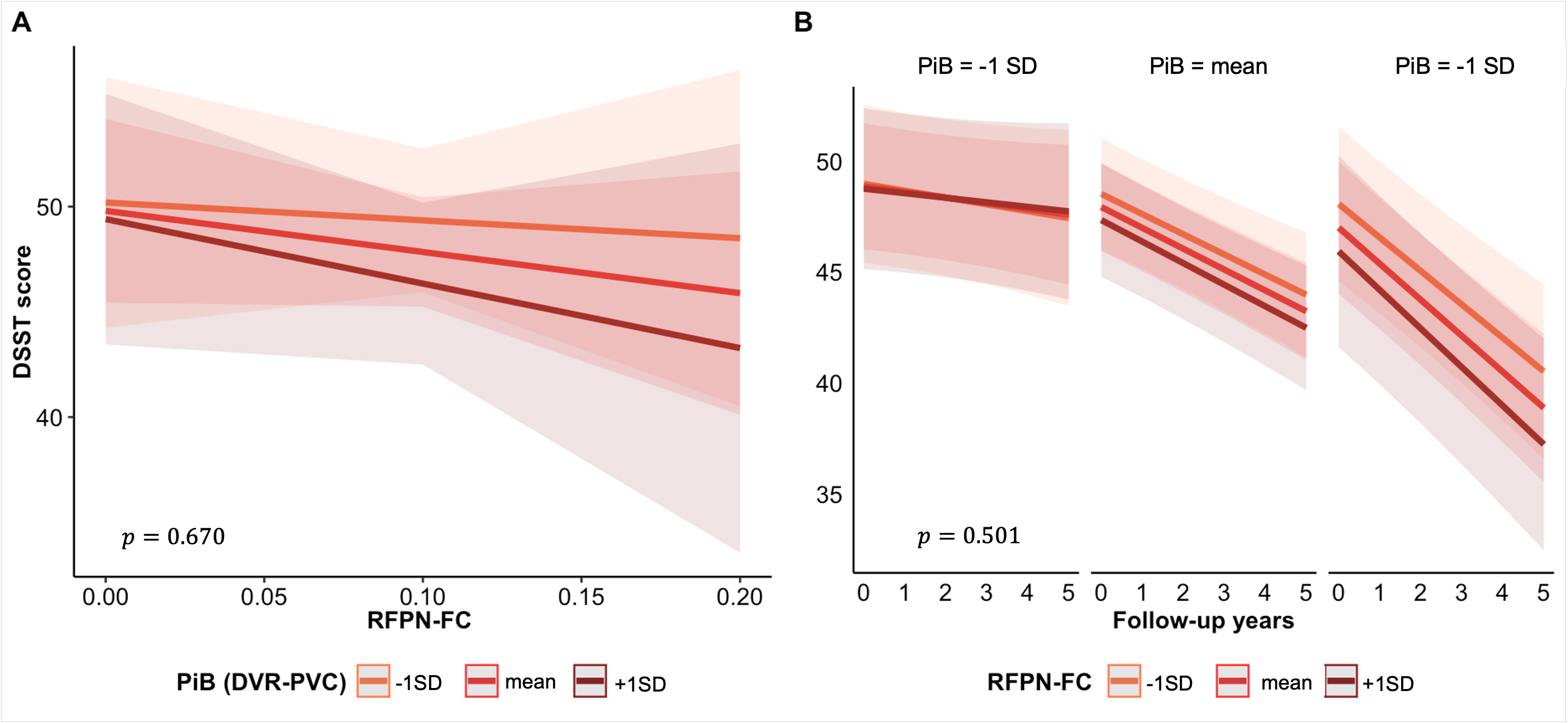

Supplement: Supplementary file 1 — Supplementary Material 1 [file 13195_2024_1485_MOESM1_ESM.tiff]

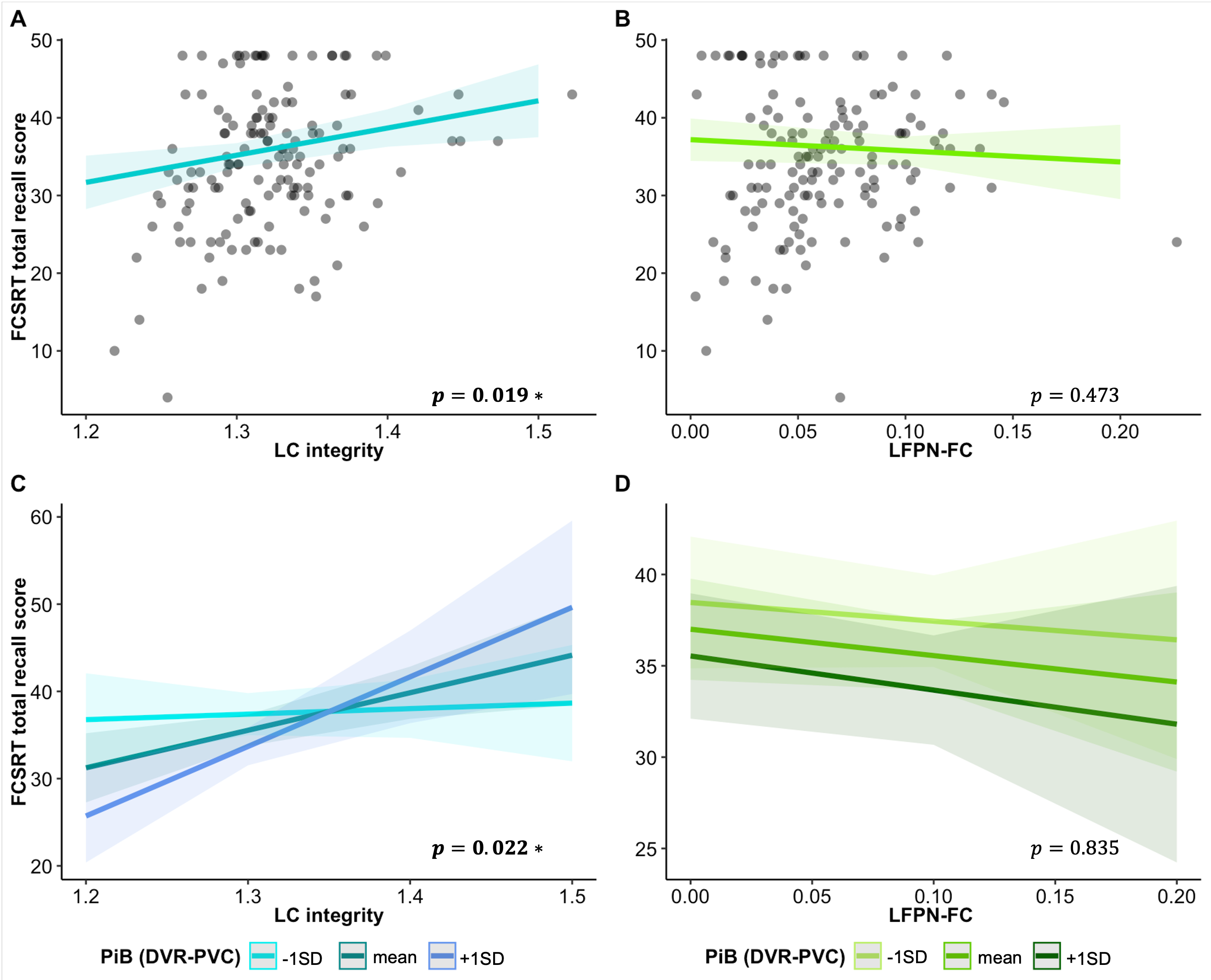

Supplement: Supplementary file 2 — Supplementary Material 2 [file 13195_2024_1485_MOESM2_ESM.tiff]

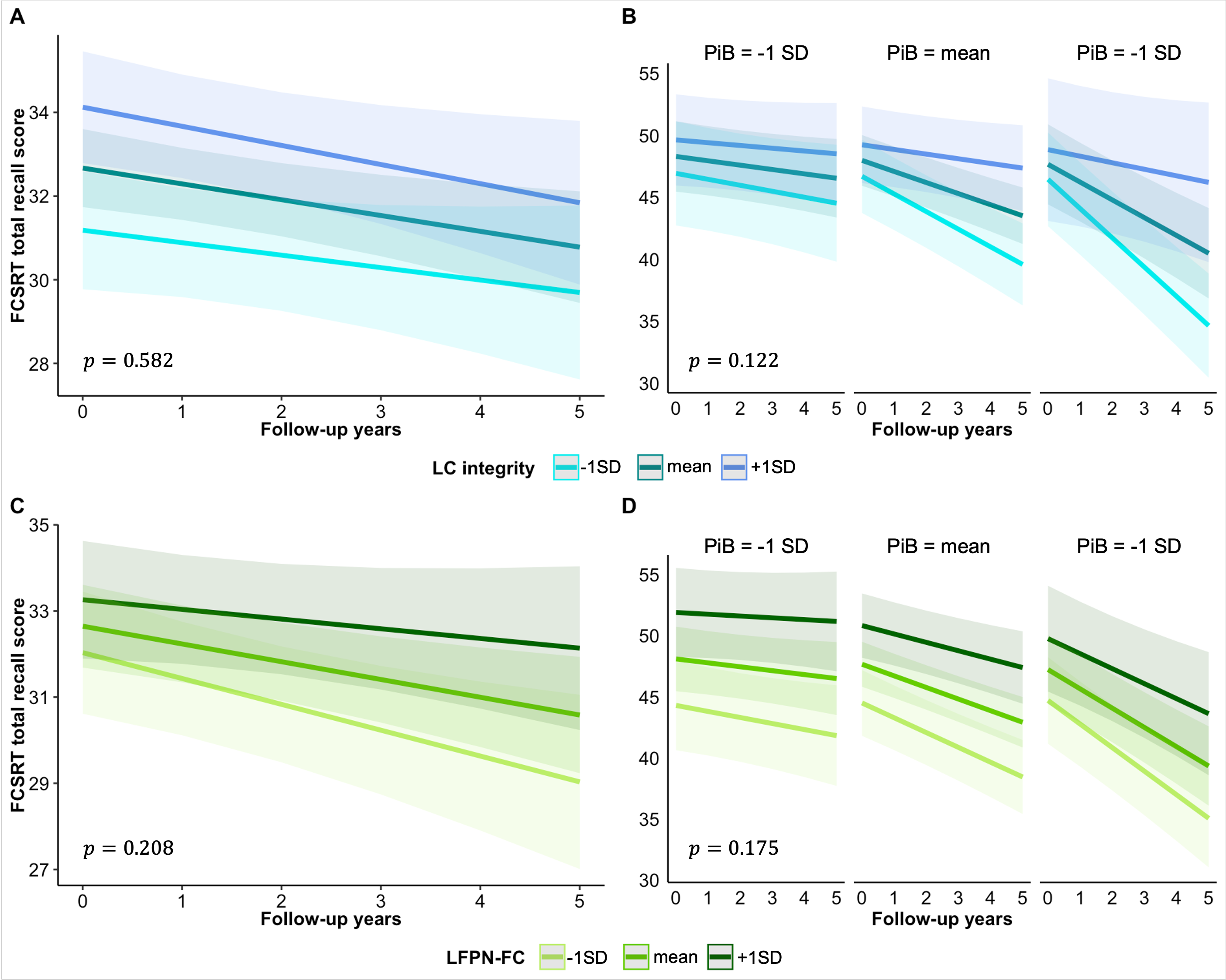

Supplement: Supplementary file 3 — Supplementary Material 3 [file 13195_2024_1485_MOESM3_ESM.tiff]

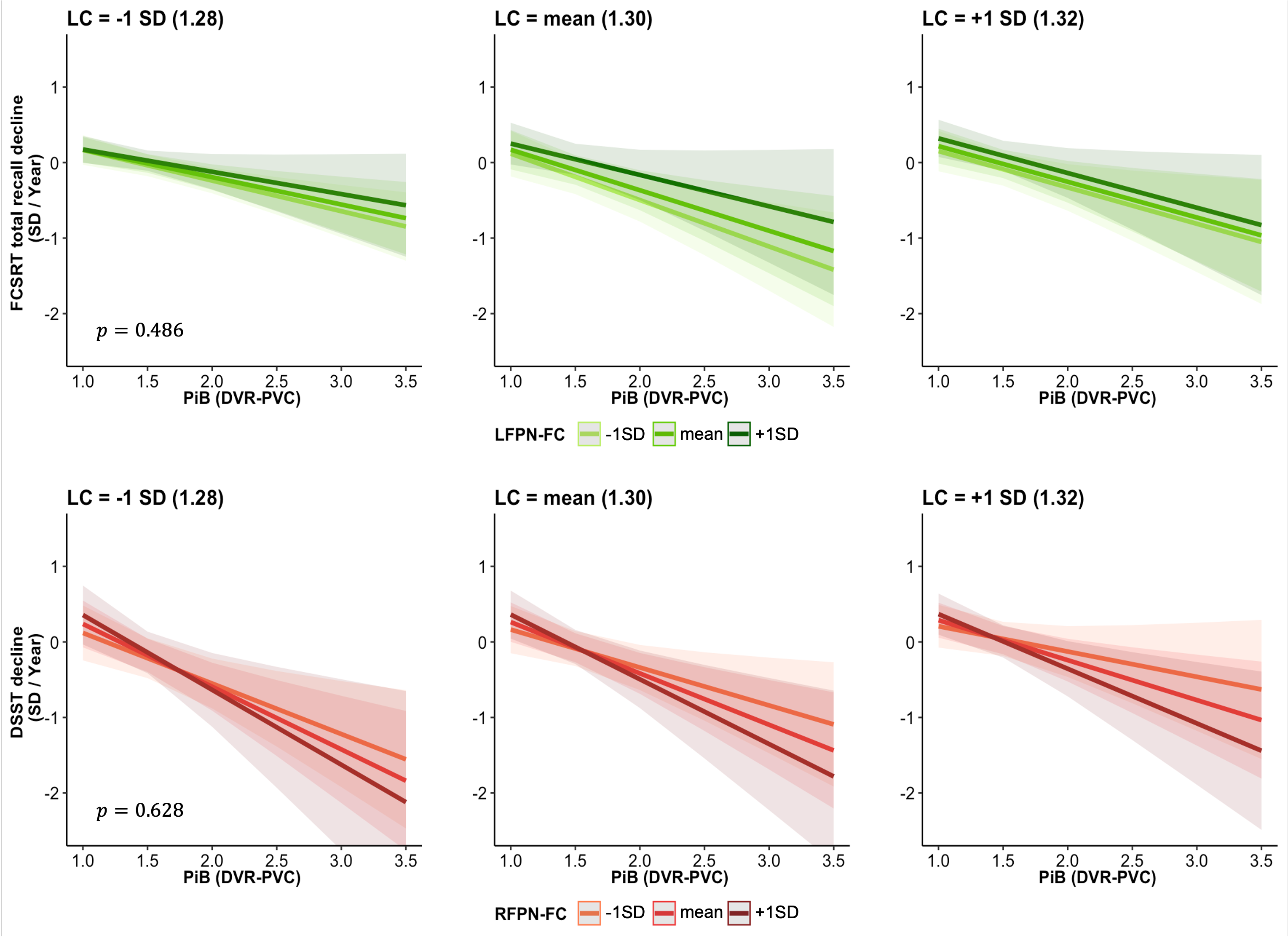

Supplement: Supplementary file 4 — Supplementary Material 4 [file 13195_2024_1485_MOESM4_ESM.tiff]
