# Supplementary material for: Locus coeruleus integrity and left frontoparietal connectivity provide resilience against attentional decline in preclinical alzheimer’s disease
Source: Alzheimers Res Ther. 2024 May 31;16:119. doi: 10.1186/s13195-024-01485-w (PMC11140954; doi:10.1186/s13195-024-01485-w)
Supplement: Supplementary file 5 — Supplementary Material 5 [file 13195_2024_1485_MOESM5_ESM.docx]

**Supplementary Material**

**Locus Coeruleus Integrity and Left Frontoparietal Connectivity Provide Resilience Against Attentional Decline in preclinical Alzheimer’s Disease**

Pahl, Jennifer^1,4^, Prokopiou, Prokopis C.^1^, Bueichekú, Elisenda^1^, Schultz, Aaron P.^3^, Papp, Kathryn V.^2,3^, Farrell, Michelle E.^3^, Rentz, Dorene M.^2,3^, Sperling, Reisa A.^2,3^, Johnson, Keith A.^2,3,5^, Jacobs, Heidi I.L.^1*^

^1^ Athinoula A. Martinos Center for Biomedical Imaging, Department of Radiology, Massachusetts General Hospital, Harvard Medical School, Boston, MA, USA.

^2^ Center for Alzheimer Research and Treatment, Department of Neurology, Brigham and Women's Hospital, Harvard Medical School, Boston, MA, USA.

^3^ Department of Neurology, Massachusetts General Hospital, Harvard Medical School, Boston, MA, USA.

^4^ Department of Neurology, University Hospital RWTH Aachen, Aachen, Germany.

^5^Gordon Center for Medical Imaging, Department of Radiology, Massachusetts General Hospital, Harvard Medical School, Boston, MA, USA.

**Figure S1:** Effect of LC integrity and LFPN-FC on FCSRT scores at baseline

*Note*. Visualization of the association between LC structural integrity on FCSRT scores (A) at different PiB levels (C) and of the association between LFPN-FC on FCSRT scores (B) at different PiB levels at baseline (D). The estimated marginal means of the interaction terms were plotted at the mean and ±1 SD for PiB load, but analyses were performed continuously. Shaded regions represent the 95% confidence interval. The units for LC integrity and LFPN-FC are arbitrary. *Abbreviations*: DVR = distribution volume ratio, FC = functional connectivity, FCSRT = free and cued selective reminding test, LC = locus coeruleus, LFPN = left frontoparietal network, PVC = partial volume corrected, PiB = Pittsburgh Compound-B, SD = standard deviation.

**Figure S2:** Effect of baseline RFPN-FC on DSST scores at baseline and follow-up

*Note*. Visualization of the control analyses using RFPN as a control network. The figure shows the association between baseline RFPN-FC on cross-sectional DSST scores (A) and between baseline RFPN-FC on DSST decline at different levels of PiB load (B). The estimated marginal means of the interaction terms were plotted at the mean and ±1 SD for PiB load, but analyses were performed continuously. Shaded regions represent the 95% confidence interval. Units of RFPN-FC are arbitrary. *Abbreviations*: DSST = Digit Symbol Substitution Test, DVR = distribution volume ratio, FC = functional connectivity, LC = locus coeruleus, PVC = partial volume corrected, PiB = Pittsburgh Compound-B, RFPN = right frontoparietal network, SD = standard deviation.

**Figure S3:** Effect of baseline LC integrity and LFPN-FC on longitudinal FCSRT performance

*Note*. Visualization of the control analyses using FCSRT as a control cognitive measure. We visualize the (A) association between baseline LC structural integrity on FCSRT scores over time (B) at different PiB levels; (C) and the association between baseline LFPN-FC on FCSRT scores over time (D) at different PiB levels. The estimated marginal means of the interaction terms were plotted at the mean and ±1 SD for PiB load, but analyses were performed continuously. Shaded regions represent the 95% confidence interval. The units for LC integrity and LFPN-FC are arbitrary*. Abbreviations*: DVR = distribution volume ratio, FC = functional connectivity, FCSRT = free and cued selective reminding test, LC = locus coeruleus, LFPN = left frontoparietal network, PVC = partial volume corrected, PiB = Pittsburgh Compound-B, SD = standard deviation.

**Figure S4:** Synergistic effect using the FCSRT as control outcome and the RFPN as control network

*Note*. Visualization of the control analyses: (A) association between LC structural integrity, LFPN-FC and PiB load at baseline on FCSRT decline over time plotted at different LC levels; (B) association between RFPN-FC, LC structural integrity and PiB load at baseline on DSST decline over time plotted at different LC levels. The estimated marginal means of the interaction terms were plotted at the mean and ±1 SD for the LC, but analyses were performed continuously. Shaded regions represent the 95% confidence interval. The units for LC integrity, RFPN-FC and LFPN-FC are arbitrary*. Abbreviations*: DSST = Digit Symbol Substitution Test, DVR = distribution volume ratio, FC = functional connectivity, FCSRT = free and cued selective reminding test, LC = locus coeruleus, LFPN = left frontoparietal network, PVC = partial volume corrected, PiB = Pittsburgh Compound-B, RFPN = right frontoparietal network, SD = standard deviation.

**Table S1:** Results of the sensitivity analyses including baseline CDR score as a covariate in our models

| ***Model*** | | | ***t-value*** | ***SE*** | ***DF*** | ***Estimate*** | ***95% CI*** | ***p-value*** |
| --- | --- | --- | --- | --- | --- | --- | --- | --- |
| Baseline | DSST | LC | 0.93 | 22.55 | 136 | 20.80 | -23.80, 65.40 | 0.358 |
|  |  | LFPN-FC | 3.17 | 28.67 | 136 | 90.78 | 34.07, 147.48 | **0.002**** |
|  |  | RFPN-FC | -0.66 | 22.54 | 136 | -14.87 | -59.45, 29.70 | 0.510 |
|  |  | LC × PiB | -0.05 | 57.17 | 134 | -3.12 | -116.18, 109.95 | 0.957 |
|  |  | LFPN × PiB | -0.43 | 61.32 | 134 | -26.56 | -147.85, 94.73 | 0.666 |
|  |  | RFPN × PiB | -0.41 | 57.42 | 134 | -23.46 | -137.02, 90.11 | 0.684 |
|  | FCSRT | LC | 2.22 | 14.77 | 136 | 32.79 | 3.59, 61.99 | **0.028*** |
|  |  | LFPN-FC | -1.21 | 20.02 | 136 | -24.13 | -63.71, 15.45 | 0.230 |
|  |  | LC × PiB | 1.42 | 36.71 | 134 | 52.10 | -20.50, 124.69 | 0.158 |
|  |  | LFPN-FC × PiB | -0.49 | 42.62 | 134 | -20.91 | -105.21, 63.39 | 0.625 |
| Longitudinal | DSST | LC | 3.37 | 3.31 | 297 | 11.18 | 4.68, 17.67 | **0.001***** |
|  |  | LFPN-FC | 1.99 | 4.18 | 297 | 8.30 | 0.11, 16.49 | **0.047*** |
|  |  | RFPN-FC | 0.22 | 3.16 | 297 | 0.70 | -5.49, 6.90 | 0.824 |
|  |  | LC × PiB | 2.48 | 7.46 | 294 | 18.48 | 3.85, 33.10 | **0.013*** |
|  |  | LFPN-FC × PiB | 0.62 | 9.10 | 293 | 5.68 | -12.15, 23.51 | 0.532 |
|  |  | RFPN-FC × PiB | -0.68 | 8.04 | 293 | -5.50 | -21.26, 10.27 | 0.494 |
|  | FCSRT | LC | -0.49 | 3.39 | 298 | -1.66 | -8.31, 4.99 | 0.625 |
|  |  | LFPN-FC | 1.26 | 4.31 | 297 | 5.42 | -3.02, 13.86 | 0.208 |
|  |  | LC × PiB | 1.62 | 7.89 | 294 | 12.78 | -2.68, 28.25 | 0.105 |
|  |  | LFPN-FC × PiB | 1.40 | 9.61 | 293 | 13.44 | -5.40, 32.28 | 0.162 |
| Synergistic | DSST slopes | LC × LFPN-FC × PiB | -2.13 | 103.58 | 130 | -220.31 | -425.24, -15.38 | **0.035*** |
|  |  | LC × RFPN-FC × PiB | -0.53 | 107.43 | 130 | -56.85 | -269.39, 155.68 | 0.598 |
|  | FCSRT slopes | LC × LFPN × PiB | -0.66 | 49.56 | 130 | -32.80 | -130.85, 65.25 | 0.509 |

*Note*: Overview about results from sensitivity analyses including baseline CDR as a covariate. Abbreviations: CDR = Clinical Dementia Rating, CI = confidence interval, DF = degrees of freedom, DSST = Digit Symbol Substitution Test, DVR = distribution volume ratio, FC = functional connectivity, LC = locus coeruleus, LFPN = left frontoparietal network, PiB = Pittsburgh Compound-B, RFPN = right frontoparietal network, SE = standard error, Estimate represents the unstandardized beta-coefficient.
